# Vibrational Energy Harvesting via Phase Modulation: Effects of Different Excitations

**DOI:** 10.3390/e28010070

**Published:** 2026-01-06

**Authors:** Paul O. Adesina, Uchechukwu E. Vincent, Olusola T. Kolebaje

**Affiliations:** 1 Department of Physics, University of Ibadan, Ibadan 200284, Nigeria; po.adesina@ui.edu.ng; 2 Department of Physical Sciences, Redeemer’s University, Ede 232101, Nigeria; 3 Department of Physics, Lancaster University, Lancaster LA1 4YB, UK; 4 Department of Physics, Adeyemi Federal University of Education, Ondo 350106, Nigeria; olusolakolebaje2008@gmail.com

**Keywords:** vibrational resonance, energy harvesting, waveforms, piezoelectric devices

## Abstract

We numerically investigate vibrational resonance (VR) and vibrational energy harvesting (VEH) in a mechanical system driven by a low-frequency periodic force, using time-periodic phase modulation of the potential function. We focus on how the characteristics of high-frequency excitations influence frequency response, power output, and harvesting efficiency. We uncover two modulation-induced phenomena—resonant induction and resonant amplification—that together produce a double VR effect. We demonstrate that in the weak low-frequency regime (ω≤0.3), the power output can exceed that of the moderate regime (ω≈1). Among the modulating waveforms, square waveform (SQW) demonstrated superior efficiency over other waveforms, which corresponds to higher response amplitude. In addition, the frequency ratio K=6.7 yielded optimal performance compared to other frequency ratios, thereby providing both maximum power output and efficiency. These findings suggest a new design strategy for energy harvesters, leveraging both primary and induced VR to enhance performance.

## 1. Introduction

The global energy crisis has intensified the demand for sustainable and reliable energy alternatives. As conventional energy sources become increasingly strained, even small contributions to energy generation are vital for promoting sustainable growth. In this context, vibrational energy harvesting (VEH) has emerged as a viable solution for converting ambient mechanical vibrations into electrical energy for powering low-power mobile or batteryless electronic devices [1,2].

Vibrational energy is one of the most prominent forms of environmental energy [3], and it can be obtained via three methods, namely, piezoelectric effect [4], electromagnetic [5], and electrostatic [6] techniques. Piezoelectric effect, is an electromechanical coupling phenomenon observed in specific crystalline materials. This property manifests as the generation of electrical charge when mechanical stress is applied to materials possessing non-centrosymmetric crystal lattices [7]. This phenomenon operates bidirectionally through two complementary mechanisms: the direct effect, wherein mechanical deformation induces electrical polarization [8] and the converse effect, involving mechanical strain generation through applied electric fields  [9]. This dual-mode operation establishes piezoelectricity as a reversible energy conversion mechanism between mechanical and electrical systems providing the theoretical foundation for numerous technological applications in sensing [10] and energy harvesting systems [11,12]. It has been empirically shown that several mechanical vibration sources can generate different magnitudes of power depending on the properties of the vibrating source. For instance, in a study by Calió et al. [13], approximately 10 mW of power was recorded from arm movements, 1 mW from typing, 100 mW from respiration, and up to 1 W while walking [13]. Consequently, different mechanical vibrations have been explored, both theoretically and experimentally. One of such techniques is the vibrational resonance (VR), proposed by Landa and McClintock [14]. VR can occur when a system is subjected to both high and low frequency periodic forces [14,15,16]. For instance, Coccolo et al. [12,17] showed that VR phenomenon in a bistable mechanical system can be used for harvesting and enhancing energy using a piezoelectric device. Electrical energy harvesting from mechanical vibration sources like heavy-duty freight railroads, cantilever with proof-mass, etc., have also been reported by some authors [18,19,20,21,22,23].

Several driving techniques have been employed to observe and control VR in nonlinear systems, such as additive time-periodic forces [24], parametric periodic forces [25,26], amplitude-modulated periodic forces [27,28,29], and phase modulation of the potential structure [30,31]. Some of these techniques have been used for VEH. For instance, an additive technique has been largely employed by Coccolo et al. [12,17], Khovanov [32], and Omoteso et al. [33] for energy harvesting and optimization, by coupling an electrical circuit to a bi-harmonically driven mechanical system via a piezoelectric device.

Here, we present a VEH method which employs high-frequency time-periodic modulation of the phase variable of a periodic structure. In contrast to additive forcing, amplitude modulation forcing, parametric perturbations, and time-delay-based resonance engineering techniques, which typically modify the parameters of the system or introduce external feedback to alter resonance conditions, the present mechanism directly regulates VR by tuning the phase variables or its structure, thereby enhancing energy harvesting without strict reliance on parameter variation, modulation or delayed feedback. Indeed, such a formalism situates the phase-modulation control technique as a complementary route within the broader spectrum of VR strategies, thereby highlighting its unique capability for active and efficient energy harvesting. The technique is based on our recent paper in which a novel double VR induced by high-frequency phase modulation was reported for a periodic structure of the Josephson junction type, thereby serving as a powerful tool for inducing and enhancing resonances [30]. Notably, phase control mechanisms are very powerful tools for controlling the dynamics of driven nonlinear systems. Several authors have previously explored these mechanisms in various contexts [34,35,36,37,38,39,40,41,42,43]. These works have demonstrated the remarkable effectiveness of different forms of phase modulation, including (i) the suppression of escapes in open dynamical systems, such as the Helmholtz oscillator [34,37]; (ii) the induction of anomalous negative mobility (ANM)—a phenomenon in which particles surprisingly move against the applied bias force [35]; (iii) the identification of specific distributions for optical control in nonlinear optical systems [38]; (iv) control of the transmission probability and conductance of a $M_{o}S_{2}$-based circuit [39,40]; (v) facilitation of the emergence of chaotic structures [41]; (vi) control of short laser pulses in plasma channels [42]; and (vii) control of the vibrational modes of trapped ions [43]. These studies highlight a few of the diverse applications of phase control mechanisms in nonlinear systems. Whereas the phase control techniques employed by Seoane et al. [34] and Coccolo et al. [37] relied on parametric perturbations, our method leverages phase-modulated high-frequency excitations to actively boost energy harvesting via induced VR. Thus, in this paper, we advance the VR technique by examining the impacts of different time-periodic phase excitations on VR and their applications to vibrational energy harvesting.

It has been shown that VR depends on the nature of the bi-harmonic forces and the ratio of their frequencies. In the study by Zhang et al. [44], it was shown that the choice of fast excitation waveform impacted the VR and pitchfork bifurcation. In this paper, we establish that the fast time-periodic phase-modulation force plays a pivotal role in governing both the VR dynamics and the energy harvesting performance, thereby impacting the output and efficiency of the harvester. This paper is organized as follows. The model and the properties of different fast excitation waveforms employed are presented in Section 2. Section 3 presents numerical results, while Section 4 draws conclusions.

## 2. Model

We have chosen for this study a mechanical system which is a modified version of the phase-excited pendulum studied theoretically and numerically by Adesina et al. [30]. In our model, we consider a direct excitation denoted by fcosωt, with the phase angle of the pendulum periodically rocked using a high-frequency time-periodic phase modulation ϕ(t) as described by Adesina et al. [30]. Unlike simple parametric modulation, such as the amplitude or frequency modulation, modulating the phase of the pendulum angle does not merely shift the timing of the pendulum’s response relative to an external reference or driving force, but it also effectively modulates the gravitational restoring force, thereby allowing the system to harness parametric resonance regimes which are highly beneficial for wide-band energy harvesting. Indeed, by assuming a small phase angle θ, such that sinθ ≈ θ and cosθ ≈ 1, the gravitational restoring force $U_{0}\sin\left[\theta +\phi \left(t\right)\right]$ can be approximated using a first-order Taylor expansion around the angular displacement θ. This yields $U_{0}\cos\phi \left(t\right)\theta +U_{0}\sin\phi \left(t\right)$, revealing that the phase modulation manifests as a dual excitation force. Specifically, it introduces an additional direct driving component $U_{0}\sin\phi \left(t\right)$ and a parametric excitation term $U_{0}\cos\phi \left(t\right)\theta$, where the latter is coupled to the system’s angular displacement θ. In practice, phase modulation of a pendulum can be implemented through a number of mechanical, electronic, and active control techniques. Most notably, periodic pivot rocking (PPR), which involves the mounting of the entire assembly on a secondary actuator, could be employed. By rotating or ”rocking” the pivot point periodically, the reference frame is shifted. This forces the pendulum to adjust its swing window, thereby introducing a time-dependent phase shift ϕ(t).

Now, assuming, arbitrarily, that a piezoelectric device (PZT) is coupled to a phase-modulated simple pendulum of mass, *m*, and length, *ℓ*, attached to a cantilever, such that it causes energy to be generated, which can be harvested via an electrical circuit coupled to it. The electrical circuit consists of a resistive load, *R*, and a capacitance, *C*. With θ being the time-varying phase angle, ϕ(t) the modulating force, and the low-frequency force denoted by fcosωt, the equation of motion reads as follows:

(1) $$ml^{2}\ddot{\theta }+\lambda \dot{\theta }+\frac{dU(\theta ,t)}{d\theta }+\mu_{v}l\;v\left(t\right)=f\cos\left(\omega t\right),$$

where U(θ,t) is the potential energy function of the pendulum and $\lambda \dot{\theta }$ is the energy dissipation to the oscillation, λ is the damping coefficient, and in the $\mu_{v}v\left(t\right)$ term, v(t) represents the voltage and $\mu_{v}$, denotes the effective electromechanical coupling coefficient of the piezoelectric device with units of torque per voltage (N.m/V). Thus, $\mu_{v}v\left(t\right)$ defines the energy transferred to the electric load *R*. The coupling equation is given as follows [12]:

(2) $$\dot{v}+\frac{v(t)}{\beta_{p}}-\mu_{c}\dot{\theta }=0,$$

where $\mu_{c}$ is the angular displacement to voltage coupling parameter, $\beta_{p}$ is the time constant of the piezoelectric dynamics which is related to the coupling capacitance *C* and to the resistive load *R* by $\beta_{p}=RC$. The potential energy function, U(θ,t), in Equation (1) is given by the following:

(3) $$U\left(\theta ,t\right)=U_{0}\left[1-\cos\left(\theta +\phi \left(t\right)\right)\right],$$

where ϕ(t) is the modulating high-frequency (HF) excitation with amplitude, *g*, frequency, Ω, and period of T=2π/Ω, while fcos(ωt) is the low-frequency (LF) driving force with frequency, ω, and amplitude, *f*.

In this study, four distinct excitation types were examined: the periodic cosine waveform (PCW), P(t); the square waveform (SQW), F(t); the symmetric sawtooth waveform (SSW), H(t); and the asymmetric sawtooth waveform (ASW), K(t), respectively, expressed as follows:

(4) P(t)=gcos(Ωt).

(5) $$F\left(t\right)=\left\{\begin{array}{cc} g, & \mathrm{for}\;0\leq t<\frac{\pi }{\Omega }\\ -g, & \mathrm{for}\;\frac{\pi }{\Omega }\leq t<\frac{2\pi }{\Omega }. \end{array}\right.$$

(6) $$H\left(t\right)=\left\{\begin{array}{cc} g\left(\frac{2\Omega t}{\pi }-1\right), & \mathrm{for}0\leq t<\frac{\pi }{\Omega }\\ -g\left(\frac{2\Omega t}{\pi }-3\right), & \mathrm{for}\;\frac{\pi }{\Omega }\leq t<\frac{2\pi }{\Omega }. \end{array}\right.$$

(7) $$K\left(t\right)=\left\{\begin{array}{cc} g\left(\frac{\Omega t}{\pi }-1\right), & \mathrm{for}\;0\leq t<\frac{2\pi }{\Omega }. \end{array}\right.$$

**Figure 1:**
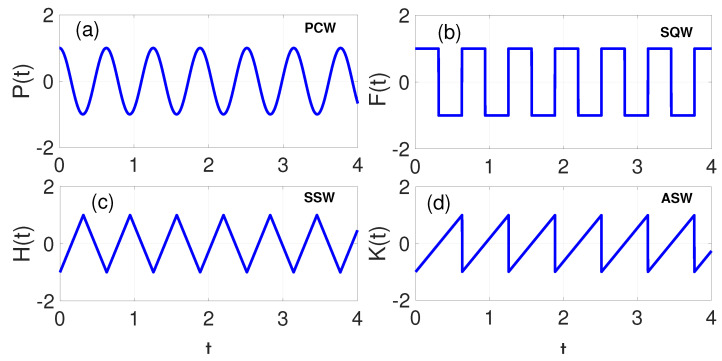
Force–time plot showing the symmetric properties of (**a**) cosine periodic waveform (PCW), (**b**) square waveform (SQW), (**c**) symmetric sawtooth waveform (SSW), and (**d**) asymmetric sawtooth waveform (ASW). The amplitude, *g*, and HF, Ω are set to 1 and 10, respectively.

The PCW, given by Equation (4) and shown in Figure 1a, is known for its smooth, continuous oscillation, demonstrating both symmetry and periodicity. It is an even function, exhibiting symmetry about the vertical axis (i.e., P(t)=P(−t)), making it remarkably useful in applications where stable and uniform oscillatory behaviour is required, such as in signal processing and vibration analysis [22]. The SQW given by Equation (5) and depicted in Figure 1b, conversely, demonstrates a clear periodicity, however, lacking smooth transitions between its crest and trough states. It oscillates between two levels with sudden/sharp transitions, similar to the P(t). Unlike the PCW P(t), the SQW is an odd function, showing symmetry about the origin (i.e., F(t)=F(−t)). This characteristic makes it an excellent choice for digital signal applications and in systems where on–off control is important. Its sharp transitions can induce higher harmonic frequencies, which can impact system dynamics significantly [45]. Furthermore, SSW exhibits linear rise and sudden drop characteristics within each period. It is given by Equation (6) and illustrated in Figure 1c. SSW exhibits symmetry about its midpoint within each period. The linear rise and rapid descent provide a unique harmonic signature that can be exploited in audio synthesis and modulation techniques. Its symmetry allows for predictable energy distribution across harmonics, making it advantageous in resonance-related investigations [22]. The ASW given by Equation (7) and illustrated in Figure 1d lacks this symmetry due to its unequal rise and fall times within each cycle. The absence of symmetry leads to a richer set of harmonics compared to its symmetric counterpart, which can be advantageous in nonlinear systems analysis or chaotic dynamics. The unique characteristics of ASW make them particularly relevant in applications where nonlinearity plays a significant role [45].

The four waveforms exhibit distinct symmetry properties which are evident in their harmonic content and coupling to the low-frequency response. Whereas the cosine waveform contains only even harmonics, the square and symmetric sawtooth waves, on the other hand, are characterized by odd harmonics due to their odd or half-wave symmetry, with the square wave’s Fourier series given by $\phi_{\mathrm{sq}}\left(t\right)=\frac{4g}{\pi }\sum_{n=1,3,5,\ldots }\frac{\sin(n\Omega t)}{n}$. Similarly, the symmetric sawtooth waveform is characterized by odd-harmonics but with distinct amplitudes, which in turn influence the phase coupling. On the contrary, the symmetry is broken by the presence of both even and odd harmonics in the asymmetric sawtooth waveform—the Fourier series being $\phi_{\mathrm{asym}}\left(t\right)=\frac{2g}{\pi }\sum_{n=1}^{\infty }\frac{\sin(n\Omega t)}{n}$—thereby introducing asymmetric forcing and energy transfer in the system’s modulated potential $U\left(\theta ,t\right)=U_{0}\left[1-\cos\left(\theta +\phi \left(t\right)\right)\right]$ [46].

## 3. Numerical Results

We now study VEH numerically using the coupled Equations (1) and (2), in order to investigate and unravel the impacts of variations in the phase modulation parameters for different HF excitations. In our numerical simulations, we employed a fourth-order Runge–Kutta method via MATLAB’s ode45 solver to integrate the coupled Equations (1) and (2) (MATLAB https://www.mathworks.com/products/matlab.html). The integration proceeded in two stages: an initial transient phase spanning $n_{1}=10$ periods with period T=2π/Ω, starting from initial conditions $Y_{0}={\left[0,0,0\right]}^{T}$, followed by a steady-state calculation phase over $n_{2}=100$ periods with fixed time step Δt=2π/(100Ω), initialized with the final state from the transient phase. Error tolerances ($\mathrm{Relative}\;\mathrm{Tolerance}=10^{-8}$, $\mathrm{Absolute}\;\mathrm{Tolerance}=10^{-10}$) were used to ensure numerical accuracy. In addition, all waveforms were implemented using the modulo operator to guarantee strict periodicity throughout the integration interval. To prevent aliasing and satisfy time-step constraints at high harmonics, we enforced Δt≤2π/(KΩ), satisfying the Nyquist criterion, where *K* is a scaling parameter. Vectorized implementations naturally preserved computational accuracy within this discretization regime for the coupled pendulum–electrical system dynamics. The response is computed from the amplitudes $Q_{s}$ and $Q_{c}$ of the Fourier spectrum of the output signal, where $Q_{s}$ and $Q_{c}$ are defined by the following [14]:

(8) $$\begin{array}{ccc} Q_{s} & = & \frac{2}{nT}\int_{0}^{nT}\theta \left(t\right)\sin\omega t\;dt\\ Q_{c} & = & \frac{2}{nT}\int_{0}^{nT}\theta \left(t\right)\cos\omega t\;dt. \end{array}$$

The system’s amplitude is given by

(9) $$A=\sqrt{Q_{s}^{2}+Q_{c}^{2}}.$$

For the LF signal, the response amplitude is given by

(10) $$Q=\frac{A}{f}=\frac{\sqrt{Q_{s}^{2}+Q_{c}^{2}}}{f}.$$

**Figure 2:**
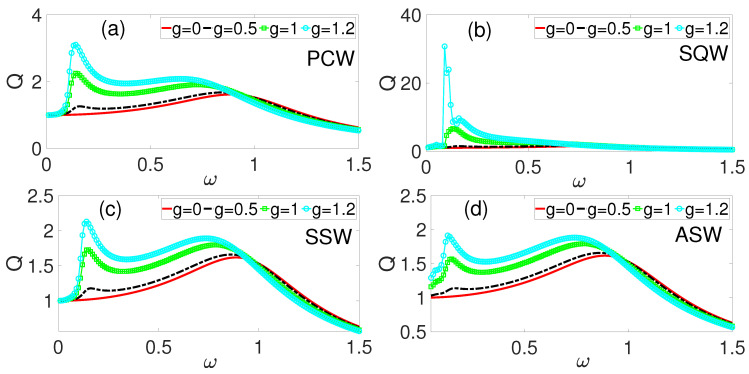
[Color Online] Frequency response amplitude, *Q* dependence on the ω for some values of phase modulation amplitude, (**a**) cosine periodic waveform (PCW), (**b**) square waveform (SQW), (**c**) symmetric sawtooth waveform (SSW), and (**d**) asymmetric sawtooth waveform (ASW).

In addition to the parameter values given in Table 1, the following values were used in our numerical calculations, except otherwise stated: $U_{0}=1.0$ and λ=0.5, f=0.1, and Ω=6.7ω. The choice of Ω=6.7ω was based on our recent paper [30], in which we found that for k=6.7, i.e., Ω=6.7ω, maximal response can be achieved, while for higher values, the response saturates for all values of *g* within the parameter range (*g* = 0 to 1.5) investigated herein. Moreover, the calculated quantities take on the following units, except otherwise stated: average power is in Watt (W) and frequencies ω and Ω are in Hertz (Hz). For the coupled oscillator (Equations (1) and (2)), we begin our discussions by exploring the impact of different kinds of high-frequency phase-modulation excitations on the frequency response amplitude, with *Q* as the function of the LF ω force. Starting with the PCW excitation already employed in our recent paper, but without coupling, Figure 2a shows the frequency response of both the zero-phase scenario (g=0) and the active phase (g>0). In the zero-phase case, a single resonance peak is observed at ω≈1. This implies that even in the zero-phase case, resonance is observed, which in this case is the primary resonance, due to the LF excitation, fcos(ωt). On the activation of phase modulation, i.e., g>0, a second resonance peak is induced in addition to the primary resonance, giving rise to double resonance. The second resonance peak appears within a low frequency regime (0.05≤ω≤0.4), where otherwise, the LF excitation could not drive the system into resonance, thereby establishing phase-modulation-induced VR in the VEH model in this regime. Here, we remark that ω=1 is in general considered moderate relative to the ω range 0.05≤ω≤1.5. Notably, with increased amplitude of the phase modulation, both the primary and the induced resonance experiences enhancement, which is the hallmark of VR. As the amplitude of the phase modulation, *g*, increases further, the induced VR underwent significant enhancement over the primary resonance. These findings, which we have reported in our recent paper [30], demonstrate that high-frequency PCW acting as a phase modulator both induces and amplifies resonance over two distinct frequency bandwidths.

We now consider other HF waveforms, with and without symmetry. Notably, the symmetry properties of the different kinds of waveforms significantly impacted the resonance behavior and energy harvesting potential of the system. As depicted in Figure 2, other waveforms shown in Figure 2b–d produce similar effects in terms of induction and enhancement of VR, but their resonance peak magnitudes vary considerably. The SQW (Figure 2b) and the PCW (Figure 2a) yielded larger responses, with the former being the highest. Comparatively, the SSW (Figure 2c) and the ASW (Figure 2d) exhibited lower response amplitudes. This variation could be attributed to the symmetry properties of these high-frequency excitations discussed in the preceding section. These findings align with the work of Ghouli and Litak [18], who observed a relationship between waveform symmetry and resonance amplitude. Very recent studies on asymmetric systems have further confirmed that asymmetric parameters significantly impact the occurrence of VR [47]. For instance, in an asymmetric electronic circuits, both the asymmetric strength arising from the systems’ components and the frequency’s influence were critical factors determining resonance behavior. The degree of asymmetricity can either enhance or suppress resonance peaks, thereby providing opportunities for the exploration and optimization of energy harvesting through careful waveform selection [7].

Proceeding further, the average power output, 〈P〉 for the harvester was computed using the following:

(11) $$\langle P\rangle =\langle \frac{v^{2}}{R}\rangle .$$

Our chosen load *R* is selected based on theoretical impedance matching ($R_{load}\approx 1/\omega C$) to approximate maximum power transfer conditions as derived in [48]. Moreover, we treated the load as an equivalent AC resistance ($R_{ac}$) denoting the rectified storage circuit. This approach was validated by Ottman et al. [49] and Liang and Liao [50], where they established that a bridge rectifier with a smoothing capacitor behaves as an equivalent linear resistance ($R_{ac}\approx R_{dc}/2$) in steady-state harmonic analysis. This approximation allowed us to capture the essential power transfer without explicitly modeling the nonlinear diode switching events.

**Figure 3:**
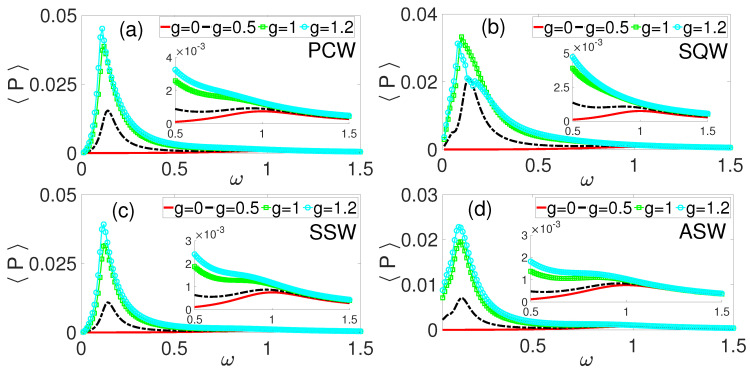
Average power harvesting, 〈P〉 as a function of ω for some values of phase modulation amplitude, *g*. (**a**) Cosine periodic waveform (PCW), (**b**) square waveform (SQW), (**c**) symmetric sawtooth waveform (SSW), and (**d**) asymmetric sawtooth waveform (ASW).

For the PCW HF excitation, the corresponding 〈P〉 is shown in Figure 3a. As the amplitude *g* increases from 0 to 1.2, electrical power is induced and enhanced in the low frequency, ω, regime, in addition to the enhancement of the power generated at ω≈1 as shown in the inset of Figure 3a. Thus, there are two frequency bandwidths available for electrical power harvesting: (i) the low frequency regime 0.5<ω<1 (as shown in the inset of Figure 3a) wherein power is induced and enhanced, and (ii) the moderate frequency regime wherein the electrical power due to the primary resonance is enhanced. These results demonstrate the effectiveness of time-periodic phase modulation as a control function for inducing and optimizing power generation by amplifying mechanical response at specific frequencies, thereby enhancing electrical power conversion efficiency [33,51]. Figure 3b–d show the average power outputs corresponding to the frequency response curves of Figure 2b–d, respectively. Comparatively, the PCW HF excitation (Figure 3a) yielded the highest average power, followed by the SSW (Figure 3c), and then the SQW HF excitation (Figure 3b), with the ASW producing the lowest power output. The harvested power at the lower frequency regime, ω<0.3 (Figure 3a–d) significantly exceeded the average power output due to the enhanced VR at ω≈1 as depicted by the inset in Figure 3. Symmetric waveforms produced more coherent VR and higher power outputs, while asymmetric waveforms introduced destructive interference, thereby reducing harvesting efficiency, similar to the recent reports in refs. [33,52]. This observation aligns with studies showing that waveform symmetry directly impacts the power density and bandwidth of vibrational energy harvesters [53,54]. The results established that increasing phase-modulation amplitude induces and enhances average power output, underscoring phase modulation’s role in optimizing energy harvesting performance.

While power output increases, evaluating the overall efficiency requires comparing harvested power to mechanical input power and considering losses in the transduction mechanism and load resistance [52]. In this regard, efficiency analysis is crucial for practical applications to ensure increased power output translates into meaningful energy gains without excessive input energy requirements. For instance, the SQW HF excitation phase modulation (Figure 2b) induced the highest frequency response magnitude, *Q*, yet the corresponding electrical power shown in Figure 3b does not depict the highest power output. This counterintuitive observation emphasizes the need for the evaluation of the vibration-to-electrical conversion rate rather than relying solely on response amplitude as a performance indicator. This could be linked to energy conversion efficiency, which is dependent on the mechanical-to-electrical transduction, electrical load optimization, and impedance matching; nonlinear saturation or suboptimal load conditions capable of causing substantial mechanical energy losses lead to weak power generation despite large oscillations [55].

The efficiency of the vibration-to-electrical energy conversion can be estimated by the energy-output to energy-input ratio [32],

(12) $$\varepsilon_{v}=\frac{P_{output}}{P_{input}}.$$

Here, the force fcos(ωt) applies a direct torque to the oscillator, doing instantaneous work at a rate proportional to the product of force and velocity. Since the potential U(θ,t) is explicitly time-dependent through ϕ(t), work is done on the system as the potential is rocked, with or without the application of an external force. The instantaneous power delivered by the external driving torque is given by

(13) $$\begin{array}{c} P_{\mathrm{torque}}\left(t\right)=f\cos\left(\omega t\right)\cdot \dot{\theta }\left(t\right). \end{array}$$

By cycle-averaging this quantity over a time interval nT, spanning multiple periods of both ω and Ω, we obtain

(14) $$\begin{array}{c} P_{\mathrm{torque}}\left(t\right)=\langle f\cos\left(\omega t\right)\dot{\theta }\left(t\right)\rangle =\frac{2}{nT}\int_{0}^{nT}f\cos\left(\omega t\right)\dot{\theta }\left(t\right)\;dt. \end{array}$$

In addition, the rate of energy change due to explicit time-dependence of U(θ,t) is as follows:

(15) $$\begin{array}{c} P_{\mathrm{mod}}\left(t\right)=\frac{\partial U(\theta ,t)}{\partial t}{|}_{\theta =\mathrm{const}}. \end{array}$$

Thus, the total cycle-averaged input power is the sum of both contributions, so that

(16) $$\begin{array}{ll} P_{input} & =P_{\mathrm{torque}}+P_{\mathrm{mod}} \end{array}$$

(17) $$\begin{array}{cc} & =\langle f\cos\left(\omega t\right)\dot{\theta }\left(t\right)\rangle +\langle \frac{\partial U(\theta ,t)}{\partial t}{|}_{\theta }\rangle . \end{array}$$

Given this input power, the efficiency is defined as follows:

(18) $$\begin{array}{c} e_{v}=\frac{P_{\mathrm{output}}}{P_{\mathrm{input}}}, \end{array}$$

where the power output, $P_{output}$ is defined as follows [32]:

(19) $$P_{\mathrm{output}}=\frac{2}{nT}\int_{0}^{nT}v^{2}\left(t\right)\;dt.$$

**Figure 4:**
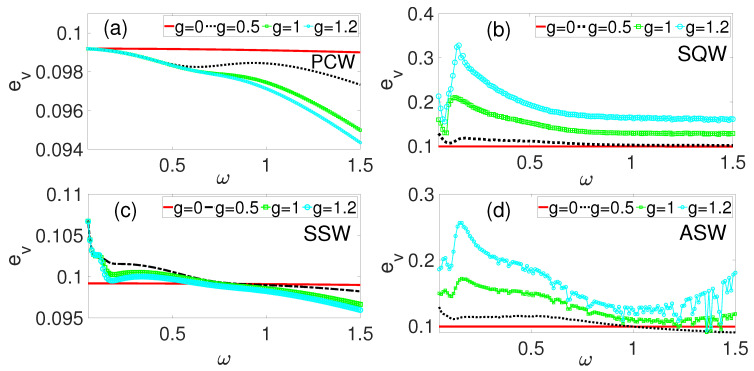
Efficiency, $e_{v}$, as a function of ω for different values of phase modulation amplitude, *g*. (**a**) Cosine periodic waveform (PCW), (**b**) square waveform (SQW), (**c**) symmetric sawtooth waveform (SSW), and (**d**) asymmetric sawtooth (ASW).

Figure 4a–d illustrate the vibration-to-energy conversion performance across different waveforms. Despite the superior performance of the periodic cosine waveform (PCW) in terms of the response amplitude (Figure 2a) and average power output (Figure 3a), Figure 4a reveals that as the phase modulation amplitude *g* increases, the efficiency is gradually suppressed across the frequency range ω. This suppression indicates that the total power input ($P_{\mathrm{input}}$) due to PCW modulation exceeds its power output ($P_{\mathrm{output}}$). In contrast, the symmetric sawtooth waveform (SSW) shown in Figure 4c demonstrates a direct correlation between *g* and efficiency. However, a critical transition occurs near ω≈1 where further increases in *g* suppress efficiency. By comparing all the waveforms at the benchmark phase modulation amplitude of g=1.2, SQW in Figure 4b exhibits superior efficiency, followed by the ASW in Figure 4d, the SSW in Figure 4c, and finally the PCW in Figure 4a, which yielded the lowest efficiency with a decreasing trend as ω increases. Higher resonance peaks, such as those in Figure 2b, correspond to enhanced vibration-to-energy conversion efficiency. The SQW’s abrupt switch between discrete levels facilitates more efficient energy harvesting compared to continuous waveforms. The symmetry characteristics and harmonic content of these high-frequency waveforms play important roles in determining the power conversion rate. The SQW’s properties, being an odd function symmetric about the origin with sharp transitions as shown in Figure 1b, generate higher harmonics which substantially enhance energy harvesting efficiency. In general, the observed improvements in resonance amplitude, power output, and efficiency via the piezoelectric mechanism highlights the effectiveness of phase-modulation in optimizing VEH.

**Figure 5:**
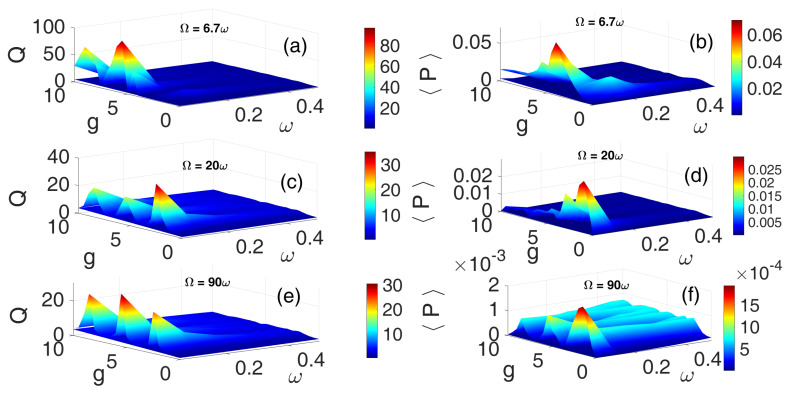
[Color Online] The dependence of the response amplitude *Q* and corresponding average power 〈P〉 on the low-frequency ω and the amplitude *g* of the PCW for three different values of frequency ratios: Ω=6.7ω (**a**,**b**), Ω=20ω (**c**,**d**), Ω=90ω (**e**,**f**). f=0.1 and λ=0.5.

In VR, the frequency relationship between the bi-harmonic excitations is critical. The resonance condition is typically expressed as Ω≫ω, and more precisely quantified as Ω=Kω, where *K* is the ratio of HF to LF. It is therefore essential to investigate how varying the frequency ratio influences the impact of different high-frequency excitations on the energy harvester’s performance. The 3D plots were obtained by scanning the two control parameters ω and *g* on uniform grids ω = 0.05:0.05:2 and g = 0:0.05:2. For each pair (ω,g), the system of ODEs was numerically integrated starting from the initial condition $Y_{0}=\left(0,0,0\right)$, as described earlier. After discarding the initial $n_{1}=10$ periods as transients, the response amplitude (*Q*) and the average power (〈P〉) over the next $n_{2}=100$ periods were computed and stored in a matrix indexed by ω and *g*. This matrix was then visualized as a 3D surface/colormap, with the horizontal axes given by ω and *g*, and the vertical axis representing the computed *Q* and 〈P〉, as appropriate. Figure 5 depicts 3D plots of the frequency response curve and the corresponding average power output for the HF PCW for three distinct frequency ratios: Ω=6.7ω (Figure 5a,b), Ω=20ω (Figure 5c,d), and Ω=90ω (Figure 5e,f). Evidently, when Ω=6.7ω, the most pronounced resonance peaks *Q* and highest average power output 〈P〉 were attained—indicating optimal VR and energy harvesting performance. In contrast, the weakest response amplitude and lowest power output were observed when Ω=90ω, as shown in Figure 5e,f. Within the parameter regime 0≤g≤10 and 0.05≤ω≤0.4, the frequency response plots reveal the presence of multiple resonance peaks across all three values of *K*. Notably, K=6.7 corresponds to a regime where phase modulation most effectively enhances system response [30] and maximizes energy harvesting under HF cosine periodic excitation.

**Figure 6:**
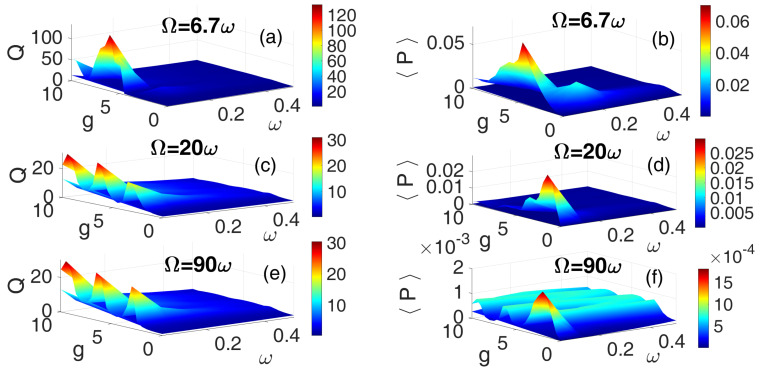
[Color Online] The dependence of the response amplitude *Q* and corresponding average power 〈P〉 on the low-frequency ω and the amplitude *g* of the SSW for three different values of frequency ratios: Ω=6.7ω (**a**,**b**), Ω=20ω (**c**,**d**), and Ω=90ω (**e**,**f**). f=0.1 and λ=0.5.

Figure 6 shows the 3D frequency response curves and the corresponding average power outputs obtained using the HF SSW for three frequency ratios: Ω=6.7ω (Figure 6a,b), Ω=20ω (Figure 6c,d), and Ω=90ω (Figure 6e,f). Consistent with earlier observations, Ω=6.7ω again yielded the most prominent resonance peaks and highest average power output, indicating optimal vibrational response and energy harvesting efficiency. Remarkably, the higher-frequency bands Ω=20ω and Ω=90ω exhibited a greater number of resonance peaks, as shown in Figure 6c,e. However, these multiple resonances do not uniformly translate into proportional power output. In particular, Figure 6d reveals that resonance peaks occurring at g>5 translates to diminished power output, suggesting that not all resonant modes contribute effectively to energy harvesting. It is noteworthy that large amplitude vibrations exhibited due to square-wave excitation do not necessarily ensure high electrical power output, as the efficiency of energy conversion is governed by mechanical–electrical transduction, electrical load optimization, and impedance matching. Indeed, nonlinear saturation or suboptimal load conditions may also lead to significant mechanical energy losses, resulting in weak power generation despite significantly pronounced resonance oscillations [55]. This clearly underscores the importance of excitation symmetry and frequency tuning in optimizing vibrational energy harvesting.

**Figure 7:**
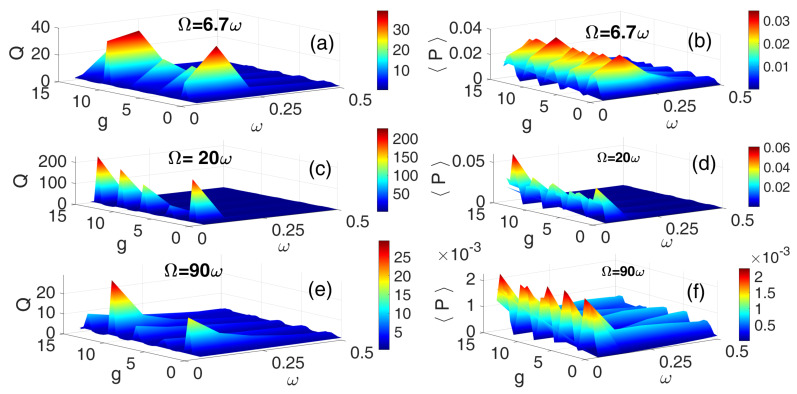
[Color Online] The dependence of the response amplitude *Q* and corresponding average power 〈P〉 on the low-frequency ω and the amplitude *g* of the HF SQW excitation for three different values of frequency ratios: Ω=6.7ω (**a**,**b**), Ω=20ω (**c**,**d**), and Ω=90ω (**e**,**f**). f=0.1 and λ=0.5.

Furthermore, Figure 7 depicts the 3D frequency response and corresponding average power output for the HF SW for three frequency ratios: Ω=6.7ω (Figure 7a,b), Ω=20ω (Figure 7c,d), and Ω=90ω (Figure 7e,f). Notably, in contrast to the results shown in Figure 5 and Figure 7c,d, the case of Ω=20ω yielded the highest resonance amplitude and average power output. Multiple resonance peaks are clearly evident across the frequency spectrum, with significant power output observed over a broad range of ω, particularly in Figure 7b, corresponding to Ω=6.7ω. This suggests that the square waveform, despite its sharp transitions, can effectively induce and sustain vibrational resonance across varying phase-modulation amplitudes, thereby enhancing energy conversion efficiency.

**Figure 8:**
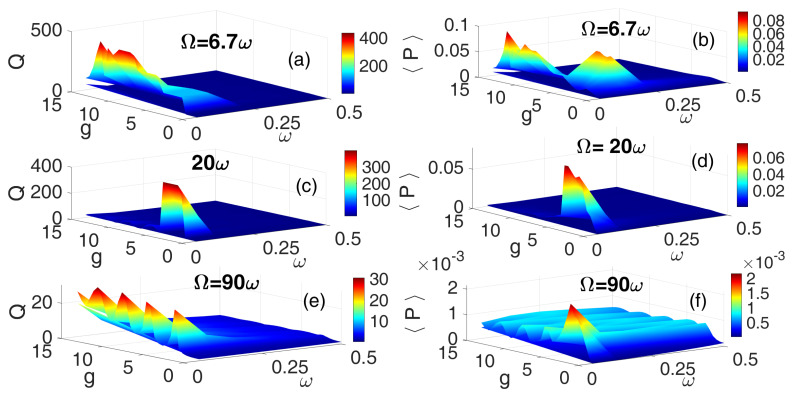
[Color Online] The dependence of the response amplitude *Q* and corresponding average power 〈P〉 on the low-frequency ω and the amplitude *g* of the HF ASW for three different values of HF: Ω=6.7ω (**a**,**b**), Ω=20ω (**c**,**d**), and Ω=90ω (**e**,**f**). f=0.1 and λ=0.5.

Finally, Figure 8 depicts the 3D frequency response and corresponding average power output using the HF asymmetric sawtooth waveform (ASW) for three frequency ratios: Ω=6.7ω (Figure 8a,b), Ω=20ω (Figure 8c,d), and Ω=90ω (Figure 8e,f). As with previous waveforms analyzed, the case of Ω=6.7ω yielded enhanced resonance peaks and higher average power output, confirming its effectiveness in inducing vibrational resonance. However, it is important to note that not all resonance peaks resulted in proportional power output. This is particularly evident in Figure 8f, where certain high-amplitude resonances do not correspond to significant energy harvesting. This highlights the influence of waveform asymmetry on the efficiency of VR conversion, suggesting that while resonance may be present, the lack of symmetry can introduce destructive interference and reduce the energy harvesting performance of the system.

## 4. Conclusions

This paper numerically investigated VR and VEH based on a simple phase-modulated mechanical system with emphasis on the roles of different kinds of excitations on the frequency response, the average power output, and efficiency. Four high-frequency excitation waveforms with frequency Ω were used as phase-modulating functions for the system driven by external periodic force fcos(ωt). Our results showed that phase modulation induced double vibrational resonance (DVR) with enhanced power output compared to the system without modulation. The induced resonance appearing at low frequency (0.05≤ω≤0.3) regimes translated to higher power output than the primary resonance near ω=1. Among the modulating waveforms, PCW demonstrated superior power output, while SQW yielded higher efficiency corresponding to higher response amplitude recorded in Figure 2b. The frequency ratio K=6.7 yielded optimal performance compared to K=20 and K=90, providing both maximum power output and efficiency. These results demonstrate that phase modulation can significantly boost energy harvesting performance through induced resonances at frequencies lower than the primary resonance, thereby offering engineers a method to optimize harvester design by exploiting both primary and induced VR phenomena for improved ambient VEH.

## Figures and Tables

**Table 1:** The simulation parameters.

Parameters	Description	Value
*m*	Pendulum mass	1 kg
*ℓ*	Pendulum length	1 m
$\mu_{v}$	Piezoelectric coupling parameter	0.0011 N.m/V
λ	Damping parameter	0.5 kg/s
$\mu_{c}$	Position to voltage coupling coefficient	4.15 $\times 10^{3}$ V·s/rad
*R*	Load resistance	300 $\times 10^{3}$ Ω
*C*	Piezoelectric coupling capacitance	$112\times 10^{-9}$ F

## Data Availability

The original contributions presented in this study are included in the article. The data are very large. Further inquiries can be directed to the corresponding author.

## Abbreviations

The following abbreviations are used in this manuscript:

VR	Vibrational resonance
DVR	Double vibrational resonance
VEH	Vibrational energy harvesting
PCW	Periodic cosine waveform
SQW	Square waveform
SSW	Symmetric sawtooth waveform
ASW	Asymmetric sawtooth waveform
